# Effects of Exogenous Cholecystokinin Octapeptide on Acquisition of Naloxone Precipitated Withdrawal Induced Conditioned Place Aversion in Rats

**DOI:** 10.1371/journal.pone.0041860

**Published:** 2012-07-27

**Authors:** Hailei Yu, Di Wen, Chunling Ma, Yanxin Meng, Shujin Li, Zhiyu Ni, Bin Cong

**Affiliations:** Department of Forensic Medicine, Hebei Medical University, Hebei Key Laboratory of Forensic Medicine, Shijiazhuang, People's Republic of China; National Institute of Agronomic Research, France

## Abstract

Cholecystokinin octapeptide (CCK-8), a gut-brain peptide, regulates a variety of physiological behavioral processes. Previously, we reported that exogenous CCK-8 attenuated morphine-induced conditioned place preference, but the possible effects of CCK-8 on aversively motivated drug seeking remained unclear. To investigate the effects of endogenous and exogenous CCK on negative components of morphine withdrawal, we evaluated the effects of CCK receptor antagonists and CCK-8 on the naloxone-precipitated withdrawal-induced conditioned place aversion (CPA). The results showed that CCK2 receptor antagonist (LY-288,513, 10 µg, i.c.v.), but not CCK1 receptor antagonist (L-364,718, 10 µg, i.c.v.), inhibited the acquisition of CPA when given prior to naloxone (0.3 mg/kg) administration in morphine-dependent rats. Similarly, CCK-8 (0.1–1 µg, i.c.v.) significantly attenuated naloxone-precipitated withdrawal-induced CPA, and this inhibitory function was blocked by co-injection with L-364,718. Microinjection of L-364,718, LY-288,513 or CCK-8 to saline pretreated rats produced neither a conditioned preference nor aversion, and the induction of CPA by CCK-8 itself after morphine pretreatments was not significant. Our study identifies a different role of CCK1 and CCK2 receptors in negative affective components of morphine abstinence and an inhibitory effect of exogenous CCK-8 on naloxone-precipitated withdrawal-induced CPA *via* CCK1 receptor.

## Introduction

Chronic use of opioids such as morphine results in the development of physical and psychological dependence, characterized by the expression of withdrawal symptoms after abstinence of drug administration or treatment of opioid receptor antagonist for precipitation. The symptoms include both physical and affective components. In animals, morphine withdrawal produces various characteristic somatic signs, such as weight loss, wet-dog shake behavior, rearing, diarrhea, and so on, and aversive avoidance behavior from the environment previously associated with morphine abstinence, which is called conditioned place aversion (CPA) [1].

Cholecystokinin (CCK), a gut-brain peptide, was initially identified as a gastrointestinal hormone, and subsequently found in the central and peripheral nervous system [2]. CCK is identified in several different sizes of the peptide including 4, 8, 33, 39, and 58 amino acid forms, and cholecystokinin octapeptide (CCK-8) is the predominant form in the CNS and acts as an anti-opioid peptide under certain circumstances [3]. The absence, in CCK receptor knock-out mice, of the regulation of CCK results in an upregulation of opioid system [4], [5]. Furthermore, there are other evidences that the CCK system modulates a variety of physiological processes [6], [7], and CCK-8 interacts with GABAergic and dopaminergic systems and plays a significant role in a wide range of activities of the central nervous system, including memory and emotional behavior [8]. The targeted mutation of the CCK receptor gene induced significant changes in the activity of the dopaminergic system [9], [10]. CCK has been shown to participate in anxiety and stress- related behaviors which reflect the negative affect of morphine withdrawal and are the most important behavioral changes involved in CPA expression [11]. Based on this profile, the CCK system may be involved in the rewarding effects of opioids and aversively motivated drug seeking process.

Several studies have investigated the role of CCK system in the development of morphine dependence and found that chronic morphine treatments enhanced the overflow of endogenous CCK in cortex, nucleus accumbens and substantia nigra [12]–[14], and CCK was necessary for the expression of morphine induced conditioned place preference (CPP) [15]. CCK2 receptor antagonists suppressed the acquisition and reinstatement of cocaine or morphine induced CPP [16], [17], as well as the aversive component of morphine abstinence through the CPA paradigm [18]. Interestingly, we have found that pretreatment with exogenous CCK-8 significantly inhibited the acquisition of morphine induced CPP [19]. This phenomenon suggested that the effect of exogenous CCK-8 was distinct from the role of endogenous CCK. Regarding the dosage, CCK-8 was able to prevent morphine dependence at high but not low concentrations [20]. Based on the pharmacological properties and specific ligand binding, two CCK receptors have been identified, CCK1 and CCK2. The expression pattern of the CCK receptors in mammals appears to be tissue specific [21]. It has been reported that the two different CCK receptors have opposing effects on the activity of dopaminergic neurons and the process of memory [22], [23]. However, there is no published study examining the effects of exogenous CCK-8 on the negative affective components of morphine withdrawal, and the subtypes of CCK receptors mediating the regulative effect of exogenous CCK-8 remains to be determined.

The present study evaluated the effects of specific CCK receptor antagonists and CCK-8 on the naloxone-precipitated withdrawal-induced CPA to clear the effects of endogenous and exogenous CCK on negative affective components of morphine withdrawal. The subtypes of CCK receptors mediating the regulative effect of CCK-8 were investigated.

## Materials and Methods

### 1 Animals

Two-hundred-and-ninety male Wistar rats were obtained from the Centre of Laboratory Animal Science at Hebei Medical University. All animals were cared for and the experimental procedures were conducted in accordance with the National Institutes of Health Guide for the Care and Use of Laboratory Animals. The rats weighed 180–200 g upon arrival in the laboratory and were habituated for 7 days prior to the experiments. All animals were housed individually. Constant temperature (21±2°C), humidity (about 60%) and a 12 h light/dark cycle (lights on at 7:00 am) were maintained throughout the experiments. Food and water were available ad libitum. All protocols in this study were approved by the Local Committee of Animal Use and Protection of Hebei Medical University.

### 2 Drugs

Morphine hydrochloride was obtained from Shenyang First Pharmaceutical Factory (Liaoning, China). Naloxone and CCK-8 were purchased from Sigma, Ltd (MA, USA). CCK-8 was suspended to concentration of 1 mg/ml in the vehicle consisting of 1% ammonia saline solution, and naloxone was dissolved in saline for a final concentration of 3 mg/ml. L-364,718, a CCK1 receptor antagonist, and LY-288,513, a CCK2 receptor antagonist were products of Tocris Cookson (Northpoint, UK), and suspended to 40 mg/ml with DMSO. Because it is generally accepted that CCK-8 does not readily penetrate the blood-brain barrier [24], CCK-8 and CCK receptor antagonists were administered centrally (i.c.v.) and not peripherally. These drug solutions were made fresh each day.

### 3 Surgery and Microinjections

The cannula was surgically implanted in an aseptic environment. According to the atlas of Paxinos and Watson (1998), rats were anesthetized with pentobarbital sodium (40 mg/kg, i.p.) and placed in a stereotaxic apparatus (Benchmark™ Stereotaxic Instruments, USA). A single hole was drilled through the skull, targeted above the left or right lateral ventricle (AP, −1.67; ML, ±0.92). A stainless steel guide cannula was implanted 3.0 mm ventrally from the surface of skull. To prevent occlusion, a dummy cannula was inserted into the guide cannula. Dental cement was used to fix the guide cannula to the skull. Animals were treated with penicillin (1000 u/day i.m.) for 3 days and allowed to recover for at least 7 days. Each microinjection was made with a 10 µl syringe (Hamilton, USA) attached to PE tubing connected to the injection cannula and given at a rate of 0.5 µl/min and a volume of 2 µl by using a syringe pump (KD Scientific, USA). The injection cannula extended 3 mm beyond the guide cannula and was left in place for 5 min following microinjections to minimize the backflow of drug solution.

### 4 Conditioned place preference apparatus

The conditioned place preference (CPP) apparatus was divided into three compartments and could be isolated by guillotine doors. Two identically sized compartments (30 cm×30 cm×40 cm) were connected by a narrower one (8 cm ×30 cm×40 cm). All three chambers were visually and tactually distinct. One large chamber was white with black horizontal stripes 2 cm wide on the walls and had a floor with stainless-steel bars (diameter 4.8 mm, placed every 1.6 cm on center), while the other large chamber was black with 2 cm wide vertical white stripes and had a floor with stainless-steel mesh (1.3×1.3 cm). The smaller middle chamber was gray and had a smooth polyvinyl chloride floor. The time spent in each compartment during a period of 15 min (900 s) was measured automatically using the Animal Video Analysis System (JLBeh soft-tech Co. Ltd. Shanghai, China).

### 5 Behavioral testing

**Figure 1:**
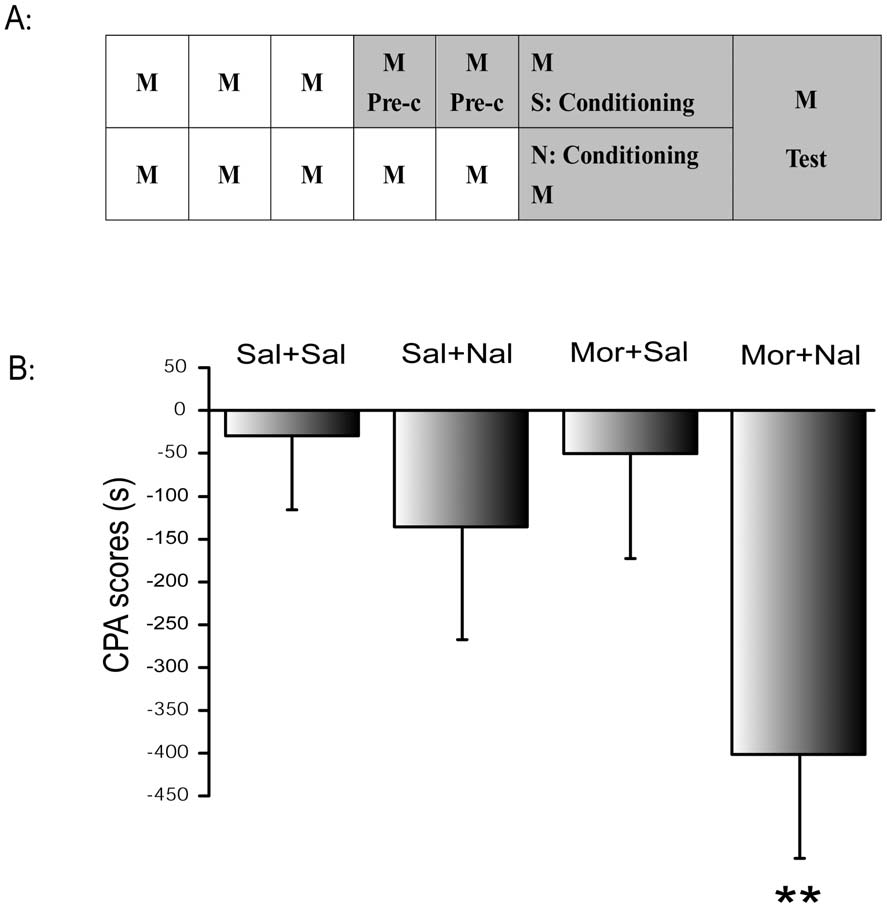
**A:** Experimental procedure of naloxone-precipitated withdrawal-induced CPA. The preference score (CPA score) defined as the time spent in the naloxone-paired compartment on day 7 (test phase) for 15 min minus the average time spent in the same compartment on day 4 and day 5 (pre-conditioning phase). Sal, saline; Mor, morphine; Nal, naloxone; **B:** Conditioned place aversion (CPA) was induced by naloxone (0.3 mg/kg, i.p.)-precipitated withdrawal in morphine (10 mg/kg, s.c.) pretreated rats. Analysis of the data obtained from CPA testing with two-way ANOVA revealed a significant morphine and naloxone effect on the CPA scores. The data are expressed as the mean ± S.D. (n = 8, 9, 8, 8). **P<0.01, compared to Sal+Sal group.

CPA consisted of three distinct phases: pre-conditioning, conditioning and testing [25]. The animals received an injection of 10 mg/kg morphine twice a day (8:00 a.m. and 20:00 p.m., s.c.) for six and a half days (from Day 1 to the morning of Day 7) to induce chronic morphine dependence (Fig. 1A).

On days 1 to 3, the animals only received morphine injections without any training. On days 4 and 5 (pre-conditioning phase), each animal was placed in the middle compartment with the guillotine doors removed at 10:00 a.m. to allow them to freely explore the three compartments for 900 s and habituate to the apparatus. The time spent in each compartment was recorded. The preferred compartment (drug-paired compartment) was determined by establishing the compartment in which the rats spent greater than 50% of the total time (450 s). Rats that spent more than 720 s on one side on day 5 or that spent more than 600 s on one side on day 4 and more than 600 s on the other side on day 5 were considered to have natural chamber bias and were excluded. This was an important step in the experimental procedure to ensure that there was no preference bias before placed condition training.

On day 6 (conditioning phase), place conditioning was performed as follows: two hours after the morphine injection (10:00 a.m.), the rats were intraperitoneally treated with saline (1 ml/kg) and confined to the saline-paired compartment for 40 mins. At 2: 00 p.m., the rats were intraperitoneally treated with 0.3 mg/kg naloxone and confined to the drug-paired compartment for 40 mins. CCK-8 or CCK receptor antagonists were intracerebroventricularly injected 15 min before the injection of naloxone.

Two hours after the last morphine treatment on day 7 (test phase), the rats were allowed to move freely in the three compartments for 900 s. The time spent in each compartment was recorded. Naloxone-precipitated withdrawal-induced CPA scores represent the time spent in the naloxone-paired compartment on day 7 (test phase) minus the average time spent in the same compartment on days 4 and 5 (pre-conditioning phase).

### 6 Experimental procedure

First, the naloxone-precipitated withdrawal-induced CPA animal model was evaluated. Rats were randomly assigned to four groups as follows: saline+saline group, saline+naloxone group, morphine+saline group and morphine+naloxone group. After establishing the animal model, three separate experiments were used in the present study. Experiment I: To examine the role of endogenous CCK system on the acquisition of naloxone-precipitated withdrawal-induced CPA, L-364,718 (10 µg), a CCK1 receptor antagonist, or LY-288,513 (10 µg), a CCK2 receptor antagonist, was given intracerebroventricularly 15 min before naloxone injection during the conditioning phase. Baseline experiments were conducted in the absence of morphine treatments. Experiment II: To observe the influence of exogenous CCK-8 on the naloxone-precipitated withdrawal-induced CPA, doses of CCK-8 (0.01, 0.1 and 1 µg) were injected 15 min prior to naloxone treatment. Subsequently, baseline experiments proceeded in the absence of opioids as well. Due to the anti-opioid characteristic of CCK-8, the induction of CPA by CCK-8 itself after morphine pretreatment was investigated. The training procedure was identical to naloxone-precipitated withdrawal-induced CPA. CCK-8 (0.01, 0.1 and 1 µg) was given 15 min before conditioned place training instead of naloxone. Experiment III: To determine whether the inhibitory effect of CCK-8 on naloxone-precipitated withdrawal-induced CPA was specific and mediated by CCK receptors, specific CCK receptor antagonists was co-treated with CCK-8. The doses of CCK-8 and CCK receptor antagonists (L-364,718 or LY-288,513) were based on previous literatures and our preliminary results.

### 7 Verification of cannula placement

After completion of the experimental sessions, rats were injected with methylene blue dye (5 µl), which was allowed to diffuse for 10 min. Then, rats were decapitated after they had been anesthetized with 20% urethane and the brains were removed and fixed with a 10% formalin solution. Gross dissection of the brain was used to verify the placement of the cannula. Only the data from those animals with dispersion of the methylene blue dye throughout the ventricles were used in the analyses.

### 8 Statistical analysis

Results of CPA score were presented as mean ± value-standard deviation (S.D.). For evaluation of the animal model of naloxone-precipitated withdrawal-induced CPA, the two-way analysis of variance (ANOVA) was used, and Bonferroni's post-hoc test was performed to assess the differences between groups. Effects of CCK-8 and CCK receptor antagonists on CPA scores were analyzed using one-way analysis of variance (ANOVA) followed by Bonferroni's post hoc test. Values of *P*<0.05 were considered statistically significant (SPSS, v. 16.0, Chicago, USA).

## Results

### 1 Histology

In the CPA tests, 42 animals were excluded because of chamber bias. At the end of behavioral experiments, the location of the cannula was checked. Due to misplacement of cannulas, 9 rats in three separate CPA experiments were not included in the statistical analyses.

### 2 The CPA model induced by naloxone-precipitated withdrawal from chronic morphine dependence

The time that morphine treated rats spent in the naloxone-paired compartment in the test session was 201±53 s, which was significantly lower than during the pre-conditioning session (603±86 s). The CPA score (i.e., the time spent in the naloxone-paired compartment in the test session minus the time spent during the pre-conditioning session) was 401±122 s, which was statistically significant compared to the saline control group (30±85 s). Analysis of the data obtained from CPA tests with two-way ANOVA revealed a significant morphine and naloxone interaction (F_1, 29_ = 9.0, *P*<0.01) on the CPA scores, and indicated that naloxone-precipitated withdrawal significantly induced CPA (Fig. 1B).

### 3 Effects of CCK receptor antagonists on naloxone-precipitated withdrawal-induced CPA

**Figure 2:**
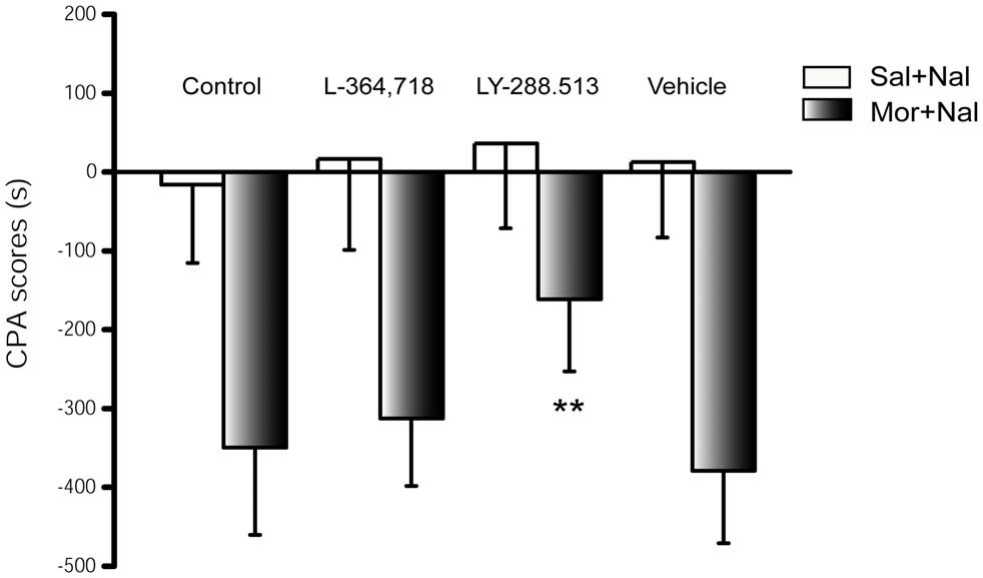
Effects of CCK receptor antagonists on the development of naloxone-precipitated withdrawal-induced CPA in morphine dependent rats. Animals were pretreated with either saline or morphine. They were subsequently microinjected with vehicle (1 µl, i.c.v.), CCK1 receptor antagonist (L-364,718, 10 µg, i.c.v.) and CCK2 receptor antagonist (LY-288,513, 10 µg, i.c.v.) 15 min prior to naloxone precipitation and CPA conditioning. Data of CPA scores are expressed as means ± S.D. (n = 10, 9, 7, 9 and 9, 8, 10, 8 in saline and morphine pretreated rats, respectively) **P<0.01 compared with the morphine pretreated and saline microinjected group by Bonferroni's post hoc test.

CCK-8 is the most potent endogenous anti-opioid peptide and its expression is up-regulated after chronic morphine treatment. CCK receptor antagonists can prevent morphine dependence in rats. Therefore, L-364,718 (10 µg), a CCK1 receptor antagonist, and LY-288,513 (10 µg), a CCK2 receptor antagonist, were examined for their ability to regulate the development of naloxone-precipitated withdrawal induced CPA in rats (Fig. 2). Baseline experiments were conducted in the absence of opioid. Intracerebroventricularly injection of L-364,718 or LY-288,513 to saline treated rats had no effect on CPA scores, and did not produce preference or aversion to any chambers (F_3, 31_ = 0.382, *P* = 0.767). Pre-treatment with LY-288,513 prior to administration of naloxone in morphine-treated rats significantly attenuated the acquisition of the naloxone-precipitated withdrawal-induced CPA (*P* = 0.001); however, L-364,718 did not have an effect on it (*P* = 1.000), when compared with the morphine-treated and vehicle-microinjected groups. As previously reported, the above-listed findings suggest that endogenous CCK is necessary for development of morphine dependence, and CCK2 receptor antagonism has a notable effect on morphine dependence.

### 4 Effects of exogenous CCK-8 on naloxone-precipitated withdrawal-induced CPA

**Figure 3:**
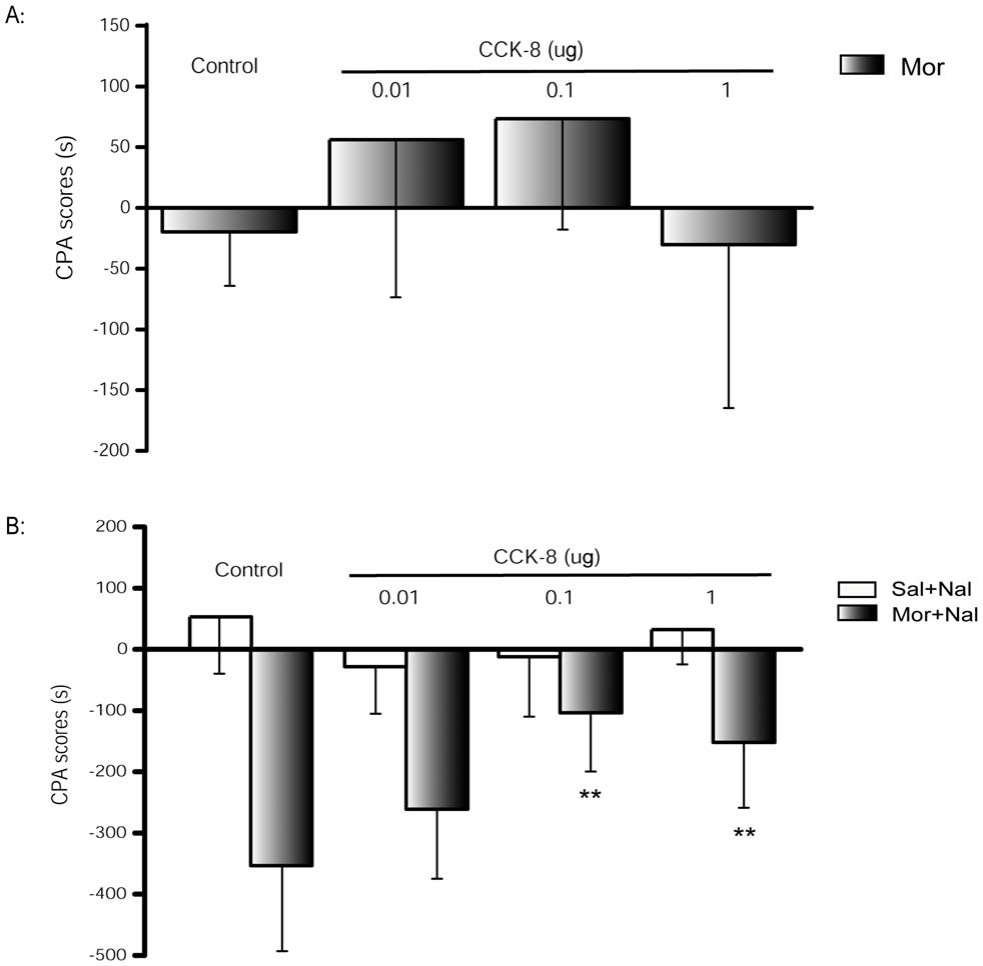
Effects of exogenous CCK-8 on the development of naloxone-precipitated withdrawal-induced CPA in morphine dependent rats. **A:** The induction of CPA by CCK-8 (0.01–1 µg, i.c.v.) itself after morphine pretreatment in rats. Animals were pretreated with morphine, and then microinjected with CCK-8 instead of naloxone for precipitation before conditioning. Data of CPA scores are expressed as means ± S.D. (n = 7, 8, 8, 9). **B:** Animals were pretreated with either saline or morphine, and then microinjected with CCK-8 (0.01, 0.1 and 1 µg, i.c.v.) 15 min before naloxone precipitation and CPA conditioning. Data of CPA scores are expressed as means ± S.D. (n = 8, 9, 8, 7 and 8, 7, 9, 8 in saline and morphine pretreated rats respectively) **P<0.01 compared with the morphine pretreated and saline microinjected group by Bonferroni's post hoc test.

Intracerebroventricular injection of exogenous CCK-8 before naloxone precipitation to morphine-treated rats attenuated the naloxone-precipitated withdrawal induced CPA (F_3, 28_ = 7.967, *P* = 0.001), indicating that exogenous CCK-8 has an identical regulatory effect on the acquisition of CPA as the antagonist (Fig. 3B). A significant inhibitory effect was observed at doses of 0.1 µg (*P* = 0.001) and 1 µg (*P* = 0.009), but not 0.01 µg (*P* = 0.771) when compared with the morphine-treated and saline-microinjected control groups. Interestingly, no dose-response effect was revealed between 0.1 µg and 1 µg CCK-8. The CPA score in 1 µg CCK-8 group was slightly higher, although it was not significant compared with the 0.1 µg CCK-8 group (*P* = 1.000). Baseline experiment was conducted in the absence of opioid as well. Microinjection of CCK-8 to saline treated rats had no effect on CPA scores (F_3, 28_ = 1.713, *P* = 0.187) and did not produce preference or aversion. Furthermore, the induction of CPA by CCK-8 itself after morphine pretreatment was investigated. CCK-8 (0.01–1 µg) could not precipitate any morphine withdrawal syndrome, and had no effect on the acquisition of CPA in morphine treated rats (F_3, 28_ = 1.904, *P* = 0.152, Fig. 3A).

### 5 Blockade of CCK1 receptor attenuates the effect of exogenous CCK-8 on naloxone-precipitated withdrawal induced CPA

**Figure 4:**
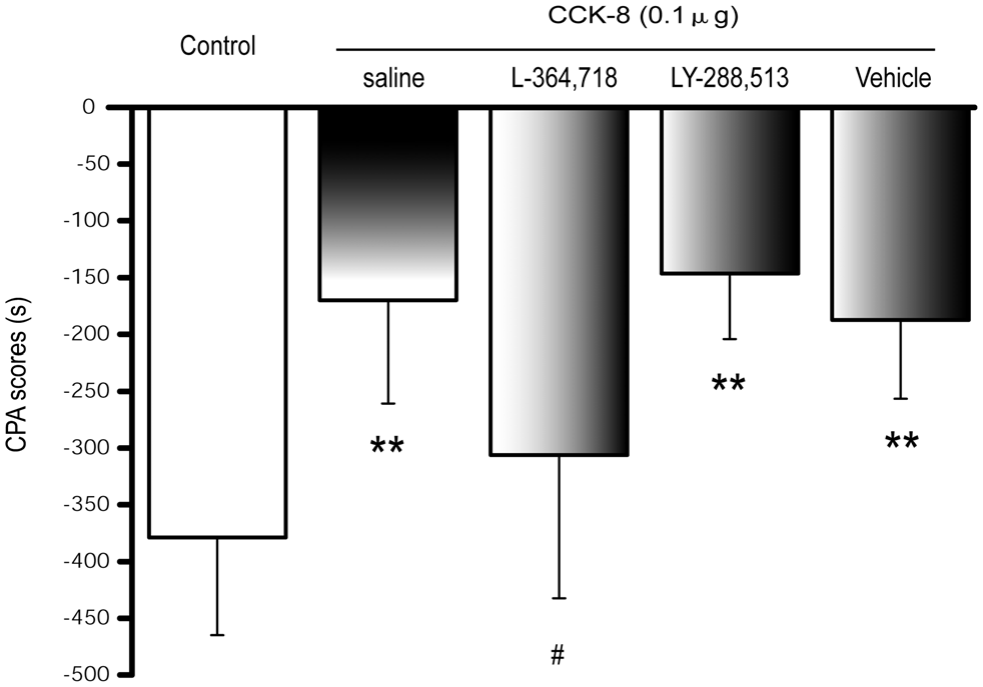
Effect of selective CCK receptor antagonists on CCK-8 inhibiting the development of naloxone-precipitated withdrawal-induced CPA in morphine dependent rats. Animals were pretreated with morphine and then co-microinjected with CCK-8 (0.01, 0.1 and 1 µg, i.c.v.) and saline, vehicle, CCK1 receptor antagonist (L-364,718, 10 µg, i.c.v.) or CCK2 receptor antagonist (LY-288,513, 10 µg, i.c.v.) 15 min before naloxone precipitation and CPA conditioning. Data of CPA scores are expressed as means ± S.D. (n = 7, 8, 9, 8, 8, respectively) **P<0.01, as compared with morphine-pretreated and saline-microinjected control, ^#^ *P*<0.05, as compared with CCK-8 plus saline co-microinjected group. Statistical analysis was conducted by one-way ANOVA followed by Bonferroni's test.

Specific CCK receptor antagonists was used to determine whether the inhibitory effect of CCK-8 on naloxone-precipitated withdrawal-induced CPA was specific and mediated by CCK receptors. We blocked CCK1 and CCK2 receptors using L-364,718 and LY-288,513, respectively, to measure the role of CCK receptor subtypes in the exogenous CCK-8 inhibition of morphine withdrawal-induced CPA (Fig. 4). Rats were co-treated with L-364,718 (10 µg) or LY-288,513 (10 µg), and CCK-8 (0.1 µg) 15 min before naloxone precipitation to induce CPA. The inhibitory effect of CCK-8 on CPA scores, was significantly reduced in L-364,718 co-treated rats, compared with no antagonist co-treated rats (*P* = 0.038). However, no change was observed in the LY-288,513 co-treated group (*P* = 1.000), indicating that the CCK1 receptor, not the CCK2 receptor, mediated the effects of exogenous CCK-8. Due to the inhibitory function of LY-288,513 and CCK-8 on CPA, as shown in Fig. 2 and Fig. 3B, the cumulative effect of LY-288,513 and exogenous CCK-8 on naloxone-precipitated withdrawal-induced CPA was not observed (*P* = 1.000).

## Discussion

Recent studies have shown the involvement of the endogenous CCK system in physical and psychological morphine dependence. It has been reported that several CCK2 receptor antagonists such as L-365,260, PD134,308 and LY-288,513, but not CCK1 receptor antagonist such as L-364,718, attenuate the development of morphine dependence and naloxone-precipitated withdrawal symptoms [26], [27]. In support of the previous studies, we found that administration of CCK2 receptor antagonist significantly attenuated naloxone-precipitated withdrawal-induced CPA in morphine pretreated rats. Similarly, CCK-8, a potent CCK receptor agonist, significantly decreased the CPA score, and systemic administration of CCK-8 inhibited the acquisition of morphine-induced CPP. These data suggest the significant effect of exogenous CCK-8 on morphine dependence, which display the same effect as the CCK receptor antagonists.

Endogenous CCK attenuates analgesia induced by opioid, thus acting as a negative feedback modulator and the most potent anti-opioid peptide. However, the induction of CPA by CCK-8 itself after morphine pretreatment was not significant, and CCK-8 could not precipitate the same morphine withdrawal syndrome as the opioid receptor antagonist. The interaction between CCK and opioids was first reported by Itoh et al. [28], and the presence of opioid receptors in CCK containing neurons suggests a potentially direct influence of opioids on CCK release [29]. Early studies failed to show an affinity of CCK for opioid receptors, indicating that CCK did not behave as a classical receptor antagonist by binding to opioid receptors. Han et al. found that CCK-8 may be capable of suppressing the high affinity opioid binding sites *via* the activation of CCK receptor [30], and then revealed that the receptor-receptor interaction between CCK2 receptors and the opioid systems may occur in an indirect manner [31]. In addition, CCK2 receptor knockout mice exhibited a hypersensitivity to the locomotor activity induced by morphine and a strongly enhanced analgesic and antidepressant-like effects of endogenous or exogenous opioids [32]–[34]. The use of highly selective receptor antagonists and antisense approaches has shown, at least in rodents, that CCK2 receptors mediate the facilitative effect of endogenous CCK in the process of morphine tolerance and dependence [35]–[37]. Above findings indicated the critical role of CCK mediated by CCK2 receptors in opioid-dependent responses.

Furthermore, the behavioral changes involved in CPA expression are thought to reflect the negative effect of morphine withdrawal, such as dysphoria, irritability and anxiety, which might contribute to aversively motivated drug seeking. Anxiety-like behavior is a prominent feature of the negative emotional consequences of naloxone-precipitated withdrawal from opioid dependence. By changing anxiety state, aversion to withdrawal during CPA training can be lessen or intensified [38]–[40]. The role for CCK in the induction and persistence of anxiety and major depression is conspicuous. Several lines of evidence suggest that CCK modulates glutamate-GABA mechanisms by acting on CCK-2 receptors, and resulting in subsequent disinhibition of the central amygdaloid nucleus and anxiety or panic-like behavior [41]–[43]. Morphine induces a potent anxiolytic-like action and CCK is acting as an endogenous antagonist of this effect of morphine [44]. Therefore, we concluded that the effect of LY-288,513 on the acquisition of CPA is at least partially related to the CCK-opioid interaction and the role of CCK in anxiety by activating the CCK2 receptor.

Taken together with our previous results, we found that the effect of exogenous CCK-8 treatments on morphine dependence was different from that of the endogenous CCK system. CCK-8 exerted a dose-dependent, biphasic effect similar to a variety of other neurally active substances, and the dose-response curve of CCK-8 was inversely U-shaped [45]. Hence, observations of exogenous CCK-8 might represent pharmacological effects, rather than physiological effects of endogenous CCK. CCK-8, the predominant centrally-active form of CCK, has high affinity for the CCK2 receptor, but low affinity for the CCK1 receptor [46]. Additionally, CCK1 and CCK2 receptors are predominantly localized to the alimentary tract and the CNS, respectively, although this distribution is not absolute as CCK1 receptors are also present in discrete regions of the CNS [47]. A previous investigation indicated that the CCK1 receptor was ineffective in the development of morphine dependence [17]. However, we found that the inhibitory effect of CCK-8 on CPA scores was significantly blocked by CCK1 receptor antagonist co-treatment. We concluded that the CCK1 and CCK2 receptors play unique and distinct roles in physiology and pathophysiology. CCK1 receptor was activated in the presence of high CCK levels, and exerted a different effect than the CCK2 receptor.

However, it is difficult to determine whether the inhibitory effects of exogenous CCK-8 are related to impairment of associative learning and memory during conditioning, or attenuation of the negative affective component of morphine abstinence. To date, the regulative effects of CCK have been demonstrated in various types of memory [48], [49]. Data showing that CCK1 receptors mediate mnemonic effects, while CCK2 receptors mediate amnestic effects have both been reported [50]. Moreover, it has been proposed that the impairing effects are due to inhibition mechanisms involving glutamate dependent neural plasticity such as long-term potentiation, which could underlie the formation of fear memory [51]. CCK-8 plays a vital modulatory role in glutamate-GABA mechanisms and facilitates long-term changes in glutamatergic synaptic transmission of the hippocampus [52], [53]. Thus, we suggest a new interpretation in which exogenous CCK-8 affects the morphine dependence by regulating the process of learning and the expression of memory during conditioning.

In summary, our study identifies a different role of CCK1 and CCK2 receptor in development of morphine dependence and an inhibitory effect of high-dose exogenous CCK-8 on the acquisition of naloxone-precipitated withdrawal-induced CPA. In addition, this study provides the first piece of evidence that the CCK1 receptor participates in the mechanism by which exogenous CCK-8 regulates the negative affective components of morphine abstinence.
